
JOURNAL INFORMATION
==============================
NLM Title Abbreviation: ScientificWorldJournal
Journal ID: ScientificWorldJournal
NLM Journal ID: 101131163
Journal ID: TSWJ

The Scientific World Journal

ISSN: 2356-6140
EISSN: 1537-744X
Publisher: Wiley

ARTICLE INFORMATION
==============================
PMCID: PMC6084179
DOI: 10.1100/tsw.2001.402
Article ID: 690968
Article version: 1
Subjects: Extended Abstract

Calcitonin Gene-Related Peptide in Tension-Type Headache

Ashina Messoud
Department of Neurology, Glostrup Hospital, University of Copenhagen and Copenhagen Headache Center, DK-2600 Glostrup, Copenhagen, Denmark
Publication date: 2001
Electronic publication date: 2001 Oct 17
Volume: 1
First page: 30
Last page: 30
Copyright: Copyright © 2001 Messoud Ashina.
Copyright year: 2001
License: This is an open access article distributed under the Creative Commons Attribution License, which permits unrestricted use, distribution, and reproduction in any medium, provided the original work is properly cited.
License URL: https://creativecommons.org/licenses/by/3.0/

==============================
4th International Meeting on Calcitonin Gene-Related Peptide (CGRP)
*TheScientificWorld* (2001) 1(S1), 30
ISSN 1532-2246; DOI 10.1100/tsw.2001.402

### CALCITONIN GENE-RELATED PEPTIDE IN TENSION-TYPE HEADACHE

Messoud. Ashina, M.D., Ph.D.
Department of Neurology, Glostrup Hospital, University of Copenhagen and Copenhagen Headache Center, DK-2600 Glostrup, Copenhagen, Denmark
E-Mail: ashina@dadlnet.dk.

In the last 10 years there has been increasing interest on the role of calcitonin gene-related peptide (CGRP) in primary headaches. Tension-type headache (muscle contraction headache) is one of the most common and important types of primary headaches and ongoing nociception from myofascial tissues may play an important role in the pathophysiology of this disorder. CGRP sensory fibers are preferentially located in the wall of arteries and nerve fibers containing CGRP accompany small blood vessels in human cranial muscles. It is well established that nociception may lead to release of CGRP from sensory nerve endings and from central terminals of sensory afferents into spinal cord. It has also been shown that density of CGRP fibers around arteries is increased in persistently inflamed muscle. These findings indicate that ongoing activity in sensory neurons in the cranial muscles may be reflected in changes of plasma levels of neuropeptides in patients with tension-type headache. To explore possible role of CGRP in tension-type headache, plasma levels of CGRP were measured in patients with chronic tension-type headache. This study showed that plasma levels of CGRP are normal in patients and largely unrelated to headache state. Findings of normal plasma CGRP add to the growing list of differences between migraine and tension-type headache. However, the findings of normal plasma levels of CGRP do not exclude that abnormalities of this neuropeptide at the neuronal or peripheral (pericranial muscles) levels play a role for the pathophysiology of tension-type headache. Investigation of CGRP in other compartments with new sensitive methods of analysis is necessary to clarify its role in tension-type headache.
